# Neuroergonomics: a review of applications to physical and cognitive work

**DOI:** 10.3389/fnhum.2013.00889

**Published:** 2013-12-23

**Authors:** Ranjana K. Mehta, Raja Parasuraman

**Affiliations:** ^1^Department of Environmental and Occupational Health, School of Rural Public Healthy, Texas A&M University, College StationTX, USA; ^2^Center of Excellence in Neuroergonomics, Technology, and Cognition, George Mason UniversityFairfax, VA, USA

**Keywords:** physical work parameters, physical fatigue, mental fatigue, vigilance, training, neuroadaptive systems

## Abstract

Neuroergonomics is an emerging science that is defined as the study of the human brain in relation to performance at work and in everyday settings. This paper provides a critical review of the neuroergonomic approach to evaluating physical and cognitive work, particularly in mobile settings. Neuroergonomics research employing mobile and immobile brain imaging techniques are discussed in the following areas of physical and cognitive work: (1) physical work parameters; (2) physical fatigue; (3) vigilance and mental fatigue; (4) training and neuroadaptive systems; and (5) assessment of concurrent physical and cognitive work. Finally, the integration of brain and body measurements in investigating workload and fatigue, in the context of mobile brain/body imaging (“MoBI”), is discussed.

## INTRODUCTION

Neuroergonomics is defined as the study of the human brain in relation to performance at work and everyday settings (Parasuraman, 2003; Parasuraman and Rizzo, 2007). It integrates theories and principles from ergonomics, neuroscience, and human factors to provide valuable insights on brain function and behavior as encountered in natural settings (Parasuraman, 2011). In this paper, we review neuroimaging techniques applicable to neuroergonomics that has expanded our understanding of the neural correlates of operators’ physical and cognitive capabilities and limitations when they interact with work systems. Moreover, while experimental laboratory studies have advanced our knowledge of brain functions during simulated work, it is important to assess operator performance in naturalistic work settings. Understanding brain function in such dynamic and mobile work settings requires the use of ambulatory neuroimaging techniques (Makeig et al., 2009).

There are two main reasons why ambulatory neuroimaging techniques need to be developed for ergonomics research and practice. First, by definition, physical ergonomics requires that participants move their limbs or bodies while carrying out some physical task. Moreover, while cognitive ergonomics studies can be conducted in immobile participants, research on *embodied cognition* has shown that cognitive processing when moving and interacting in the physical world may have unique characteristics that can only be captured with mobile neuroimaging (Clark, 1998; Parasuraman, 2003; Raz et al., 2005). This review discusses the use of neuroergonomics methods to evaluate brain responses in mobile work environments. We discuss the suitability and feasibility of mobile and immobile brain imaging techniques in the context of physical neuroergonomics, cognitive neuroergonomics, and neuroergonomic assessment of concurrent physical and mental work. Finally, we consider the requirements and utility of combined brain and body measurements in investigating workload and fatigue for neuroergonomic investigations.

## NEUROERGONOMIC METHODS

Neuroergonomic studies rely heavily on existing neuroimaging techniques to understand brain structures, mechanisms, and functions during work. Neuroimaging techniques applicable to neuroergonomics fall into two general categories, those that are direct indicators of neuronal activity in response to stimuli, such as electroencephalography (EEG) and event-related potentials (ERPs), and those that provide indirect metabolic indicators of neuronal activity, such as functional magnetic resonance imaging (fMRI), positron emission tomography (PET), and functional near infrared spectroscopy (fNIRS). EEG represents summated post-synaptic electrical activity of neurons firing in response to motor/cognitive stimuli as recorded on the scalp, and thus offers excellent temporal resolution of electromagnetic brain changes, on the order of milliseconds. In comparison, fMRI and PET techniques, that provide information on cerebral blood flow in response to neuronal activity, have low temporal resolution (on the order of about 10 s), but offer excellent spatial resolution (1 cm or better) and unlike EEG, they provide valuable information on location of the neural signal generated.

**FIGURE 1:**
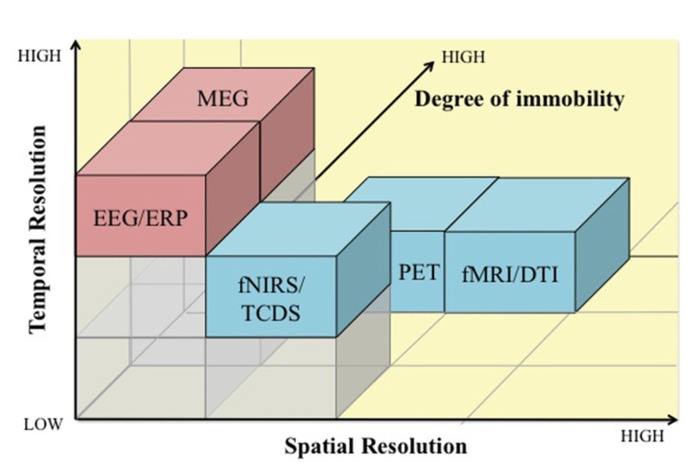
**A comparison of electromagnetic (pink) and hemodynamic (blue) neuroimaging techniques for use in neuroergonomics based on temporal resolution (*x*-axis), spatial resolution (*y*-axis), and degree of immobility (*z*-axis).** EEG, electroencephalography; ERP, event-related potentials; MEG, magnetoencephalography; fNIRS, functional near infrared spectroscopy; TCDS, transcranial Doppler sonography; fMRI, functional magnetic resonance imaging; DTI, Diffusion tensor imaging; PET, positron emission tomography.

Since neuroergonomics distinguishes itself from traditional neuroscience in that it evaluates brain functions in response to work, it is important that the neuroergonomic methods provide the flexibility to assess brain function in naturalistic work settings. Some neuroimaging techniques are better designed for and adapted for assessing brain functions in mobile work environments than others. The pros and cons of neuroergonomic methods are discussed in reference to three criteria: (1) temporal resolution, (2) spatial resolution, and (3) degree of immobility. **Figure 1** illustrates how these neuroimaging techniques compare against each other based on the three criteria. In addition, **Table 1** lists these methods and their major characteristics, such as portability, cost, along with spatial and temporal resolution. In this section, we provide a brief review of the various methods that have been used in neuroergonomic evaluations of human work, emphasizing measures of brain function and applicability in mobile experimental/field settings.

**Table 1:** List of neuroergonomic techniques and their major features.

Method	Measures/stimulates	Portability/mobility	Cost	Spatial resolution	Temporal resolution
MRI	Gray matter volume	None	High	High	NA
DTI	White matter integrity	None	High	High	NA
fMRI	Relative blood oxygenation	None	High	High	Low
fNIRS	Oxyhemoglobin and deoxyhemoglobin	High	Low	Moderate	Low
TCDS	Cerebral blood flow velocity	Moderate	Low	Low	Low
EEG	Summated post-synaptic electrical activity	Moderate	Low	Low	High
ERP	Stimulus or response-related electrical activity	Moderate	Low	Low	High
TMS	Brain activation or inhibition	Low	Moderate	High	High
tDCS	Brain activation or inhibition	High	Very low	Low	Low

Electroencephalography signals are the spatial summation of current density induced by synchronized post-synaptic potentials occurring in large clusters of neurons measured at the scalp (Pizzagalli, 2007). The EEG is recorded as differences in voltage between active electrodes at different positions on the scalp, such as the frontal, parietal, temporal, and occipital lobes of the brain according to the International 10–20 System, and a reference electrode, typically the ear. EEG signals comprises of different frequency bands, each associated with various cognitive and physical states. Spectral analyses of EEG signals can be conducted to assess power in different frequency bands: delta (0.5–3 Hz), theta (4–8 Hz), alpha (8–13 Hz), beta (13–30 Hz), and gamma (40–50 Hz). Another commonly computed EEG-driven spectral metric (i.e., brain) used in conjunction with muscular output (i.e., body) is corticomuscular coherence (CMC). CMC reflects “communications” between the brain and muscle and is determined as the coherence between sensorimotor cortex activation obtained from EEG and muscular activation as measured by electromyography (EMG) during motor activities (Halliday et al., 1995).

Electroencephalography-derived ERPs represent the brain’s neural response to specific sensory, motor, and cognitive events. ERPs represent the outcome of signal averaging of EEG epochs time-locked to a particular stimulus or response event. To evaluate mental workload or examine human error (Fedota and Parasuraman, 2010), ERP waveforms are examined for changes in the amplitude and latency of different ERP *components*, typically defined as positive or negative peak activity (such as the P3 and N1 components) or slowly rising activity such as the lateralized readiness potential (Luck, 2005). To assess neural bases of motor activities, motor-related cortical potential (MRCP) ERP components have been studied that are characterized by a slowly rising negative potential, called *Bereitschaftspotential* (BP) or readiness potential, which is followed by a sharp rising negative potential, known as negative slope. As the onset of MRCP occurs prior to the onset of the motor activity, MRCP is considered to indicate pre-motor activity, which involves specific brain regions that prepare for a desired motor behavior (Kornhuber and Deecke, 1965).

Electroencephalography-driven metrics, both spectral and temporal, in evaluating brain function during naturalistic complex tasks are relatively unobtrusive so that it does not interfere with the operator’s work performance. Its compact size and low cost, compared to other neuroimaging techniques such as fMRI and PET, makes it fairly well suited for use in both laboratory and field conditions. While artifacts attributed to movement, eye blinks, and physiological interference accompany EEG data, several algorithms have been developed to allow for the removal of noise in the EEG signal in real time or during post processing of the data (Jung et al., 2000). Recent developments in making “field-friendly” EEG systems include “dry” electrode caps, which do not need extensive participant preparation time, as well as wireless systems that do not require the participant to be tethered to cables. These technical developments have enhanced the relevance and value of EEG for mobile applications (Makeig et al., 2009).

Cerebral hemodynamic techniques such as fMRI and PET provide valuable information on source locations of distinct neural activation patterns associated with simple and complex cognitive, motor, and affective functions. While PET uses injected radioactive tracers to measure the blood flow in response to stimuli, based on their respective magnetic characteristics fMRI focuses on the resulting contrast between oxygenated and deoxygenated blood called the Blood Oxygenation Level Dependent or BOLD signal (Poldrack et al., 2011). Both fMRI and PET have been fundamental in advancing our knowledge on brain functions and mechanisms during simple, and relatively static, cognitive and motor tasks. By leveraging high spatial resolution offered by fMRI measurements, reliable techniques for the fMRI-EEG integration have been made possible that offer greater spatio-temporal resolution of imaging dynamic brain activity as well as significant improvement over the conventional fMRI-weighted EEG source imaging techniques (Liu and He, 2008; Yang et al., 2010). At the same time, fMRI and PET present several limitations in studying brain functions, such as the required supine position that may yield altered hemodynamic changes than seated or standing positions (Raz et al., 2005), limited mobility, and restrictions on synchronized brain-body measurements (Makeig et al., 2009). Moreover, the increasing need to examine brain activation patterns in complex tasks more representative of natural everyday situations have led researchers to adopt alternative neuroimaging techniques that offer better mobility features.

Functional near infrared spectroscopy is a non-invasive optical technique for measuring cerebral hemodynamics similar to PET and fMRI but with lower spatial resolution. By utilizing the tight neurovascular coupling between neuronal activity and regional cerebral blood flow (Villringer and Chance, 1997) fNIRS measures regional cerebral hemodynamic changes (i.e., changes in oxy- and deoxy-hemoglobin levels) (Jobsis, 1977). Since oxygenated and deoxygenated blood can be contrasted by their different optical absorption properties, fNIRS detects the levels of these blood parameters in response to neuronal activity. fNIRS is portable, inexpensive, and has shown to be an effective tool in quantifying cortical activation during static and dynamic motor movements, without causing substantial movement artifact issues (Perrey, 2008). While fNIRS measurements, particularly oxygenated hemoglobin levels, have shown to be strongly correlated to the fMRI BOLD signals, albeit with relatively lower signal to noise ratio (Strangman et al., 2002; Cui et al., 2011), unlike fMRI and PET its effectiveness in mapping neural activations across closely connected regions or within deep cortical areas is limited due to its relatively lower spatial resolution. Multimodal imaging approaches using both fNIRS and EEG systems have demonstrated that fNIRS is capable of enhancing event-related desynchronization-based EEG measurements significantly (Leamy et al., 2011; Fazli et al., 2012).

While fNIRS enables measurement of oxygenated and deoxygenated hemoglobin levels in cortical regions, transcranial Doppler sonography (TCDS) uses ultrasound to image cerebral blood flow to the brain hemispheres (Aaslid, 1986). TCDS uses an emitter attached to the head to direct ultrasound toward the middle cerebral artery (MCA) within the brain, and a receiver then records the frequency of the sound wave reflected by red blood cells moving through the artery. The magnitude of the change in frequency (the Doppler shift) varies directly proportional to the velocity of blood flow within the artery (Duschek and Schandry, 2003). In response to increased task-related neuronal activity, MCA blood flow velocity increases to remove by-products of the metabolic exchange, which is captured using TCDS (Aaslid, 1986). TCDS has become increasingly popular in cognitive neuroergonomic studies of vigilance and mental workload (Warm and Parasuraman, 2007). However, because cerebral blood volume and blood flow velocity is influenced by systemic changes such as heart rate and blood pressure during exercise (Ainslie et al., 2007), TCDS is less popular in assessing task-related neuronal activity in physical neuroergonomic studies of fatigue.

In contrast to the excellent temporal resolution offered by EEG techniques (on the order of milliseconds), magnetic resonance imaging (MRI) provides a structural image of the brain and offers excellent spatial visualization of deep internal parts, such as the hippocampus. While MRI provides static images of the brain that is critical in examining structural changes in the brain due to diseases (such as tumor), its application in studying structural changes in the brain over time (i.e., plasticity) has provided important information on learning and training (Huttenlocher, 2002). A relatively newer MRI technique, called diffusion tensor imaging (DTI), uses MRI to target the diffusion of water molecules in the axons that make up white matter in the brain and allows for the computation of *fractional anisotrophy* (FA). FA values can range from 0 to 1, where 0 indicates non-directional (isotropic) and 1 indicates perfectly directional (anisotropic) diffusion. Higher FA values are thought to reflect greater integrity of white matter linking different cortical and subcortical regions of the brain. Several recent studies have assessed the effectiveness of cognitive and motor training on white matter integrity using the DTI technique (Draganski et al., 2004; Takeuchi et al., 2010; Strenziok et al., 2014). In general, the MRI technique does not offer any mobility features, but an MRI static image can be overlaid with more dynamic fMRI images (i.e., blood oxygenation) so that areas of activation can be associated with particular brain regions.

The electromagnetic and hemodynamic neuroimaging techniques discussed thus far are based on sensing brain activity while a human operator is engaged in cognitive or physical work. As such, all such techniques are *correlational*, thus it may be difficult to establish *causal* links between brain activity and performance using these methods. Researchers have therefore turned to non-invasive stimulation techniques that modulate brain activity, such as transcranial magnetic stimulation (TMS) and transcranial direct current stimulation (tDCS), in order to establish such causal associations. These techniques allow for temporary inhibition or activation of specific brain regions thereby allowing researchers to examine the causal role of different brain regions in various cognitive functions (Walsh and Pascual-Leone, 2005). TMS and tDCS can also be used to modulate brain activity so that the performance of a given cognitive or motor task is improved (Coffman et al., 2014). Alternatively, these techniques can also be applied not to enhance performance over baseline, but to reduce or eradicate a normally occurring performance limitation, such as performance decrements that occur in vigilance tasks (Nelson et al., 2014).

Transcranial magnetic stimulation uses a magnetic coil that is positioned over the participant’s scalp over a brain region of interest to send electrical current that changes the magnetic field perpendicular to the head. This induces current flow in the underlying cortical issue, sufficient to alter neural firing (Walsh and Pascual-Leone, 2005). The spatial resolution of TMS is relatively high, particularly when the participant’s MRI scan is available to guide the TMS coil placement. The temporal resolution is also high, since the TMS pulses can be delivered with millisecond precision. However, due to the equipment setup TMS does not offer a sufficient degree of mobility needed for neuroergonomic assessment in naturalistic work settings. Whereas TMS uses changing magnetic pulses, tDCS uses small DC electric current (1 or 2 mA) with electrodes attached to the scalp. A positive polarity (anode) is typically used to stimulate neuronal function and enhance performance, and a negative polarity (cathode) is used to inhibit neuronal activity. Compared to TMS, tDCS has low spatial and temporal resolution, but has the advantage that it is portable and very inexpensive and thus is more likely to be adopted in applied neuroergonomic studies.

## PHYSICAL WORK

Ergonomics began as the science of work to maximize productivity, particularly in physical work environments, but has since then expanded to become a scientific discipline concerned with the understanding of the interactions among humans and other elements of a system, in order to optimize human well-being and overall system performance. Physical ergonomics focuses on human physical capabilities and limitations, pertaining to anthropometry, physiology, and biomechanics of the human body, as they relate to physical work (Karwowski et al., 2003). Traditional ergonomic evaluations focus solely on peripheral outcomes, such as force or muscle activity, and disregard the contributions of the brain during work. Physical neuroergonomics is an emerging field of study that focuses on the knowledge of human brain activities in relation to the control and design of physical tasks (Karwowski et al., 2003), by taking into consideration an operator’s physical, cognitive, and affective capabilities and limitations. Here we consider how neuroergonomic methods have been employed to evaluate different physical work parameters (such as force production and repetition) and physical fatigue (localized muscle fatigue and whole body fatigue).

### PHYSICAL WORK PARAMETERS

The primary goal of ergonomics is to ensure that work demands are always lower than operator capacity, and the conventional assessment of work demands include measuring biomechanical and physiological outcomes, such as joint torque, muscle activity, and heart rate, in laboratory and field settings. There has been recent interest in assessing physical work using neuroergonomic methods in controlled laboratory conditions; however, there is a clear lack of neuroergonomic studies in assessing physical work in actual field/work settings. Like any new field, physical neuroergonomics research first needs to understand the capabilities, limitations, and considerations of existing neuroimaging techniques on simulated work environments that can help build the knowledge base necessary to perform research in naturalistic work environments.

Since physical work can involve both static and dynamic work at different intensities, repetitions, and durations, which in turn can affect autonomic responses, different work parameters can influence the type of measurement technique adopted. For example, dynamic or ambulatory tasks, such as walking or lifting, cannot be assessed using fMRI due to mobility constraints. More appropriate neuroimaging methods to evaluate ambulatory physical work are EEG, ERP, and fNIRS. Of these, EEG appears to be the most common neuroimaging technique since it provides excellent temporal resolution. Effective artifact removal techniques are available that allow for its use in evaluating dynamic tasks. For example, EEG-derived MRCP has provided valuable information on the role of cortical motor commands (represented by the MRCP) on the control of voluntary muscle activation. MRCP from the supplementary motor area and the contralateral sensorimotor cortex has shown to be highly correlated with force production and rate of force production during isometric elbow-flexion, and associated muscle activity (Siemionow et al., 2000). Of note, a recent fNIRS investigation has demonstrated obesity-related alterations in neural patterns of force control (i.e., lower prefrontal cortex activation associated with decreased joint stability) that can shed some light on the increased incidence of injury rates and higher work absenteeism in obese workers (Schulte et al., 2007; Mehta and Shortz, 2013). High repetition is one of the major work-related risk factors that contribute to the development of musculoskeletal disorders (Bernard, 1997). To evaluate the effects of repetition that involves flexion and extension of a joint, traditional ergonomic methods focus on muscular responses such as EMG. In a study investigating thumb flexion and extension movements, EEG-derived MRCP findings from the supplementary motor area and contralateral motor cortex demonstrated that extension and flexion result from separate corticospinal projections to the motor neurons (Yue et al., 2000). Thumb extensions resulted in lower EMG but elicited greater brain responses than flexion movements. This particular finding may be important to our understanding of the etiology of musculoskeletal disorders due to repetitive motion. Real work environments are seldom static, and can require operators to focus not only on the physical work demands but also on the necessary visual/auditory cues associated with the tasks. Such tasks, which are dynamic and require visuomotor control, have shown to increase corticomuscular coupling at higher EEG frequencies (i.e., gamma bands), indicating the adaptive role of cortical oscillations in rapidly integrating visual (or new) information with the somatosensory information (Marsden et al., 2000; Omlor et al., 2007). These findings have important implications for task analysis and design, particularly for work tasks that require visual feedback or fine or precise control of body motions.

### PHYSICAL FATIGUE

Fatigue is defined as the inability to maintain required power after prolonged use of the muscle(s) (Latash et al., 2003), and can be affected by central (i.e., motivation, cortical activity, etc.) and peripheral (i.e., changes in muscle contractile properties) mechanisms. Neuroergonomic methods can help examine the role of central brain mechanisms in fatigue development. Based on the work tasks, fatigue in the workplace can be broadly categorized as localized muscle fatigue, which is the fatigue of specific muscle groups during tasks such as assembly line work or precision work, and whole body fatigue, which is more cardiorespiratory in nature that can occur during manual materials handling tasks. Commonly used ergonomic indicators of localized muscle fatigue include a reduction in force generating capacity (Vøllestad, 1997) and a decrease in EMG power spectrum (Mehta and Agnew, 2012). However these measures do not delineate the contributions of central fatigue from peripheral fatigue. Using EEG-derived MRCP, Johnston et al. (2001) demonstrated a significant increase in the activity of the BP component and the motor potential (MP) component of the MRCP, associated with a decline in force production and reduced EMG activity during a fatiguing grasping task. These increases in the early components of MRCP may reflect development of compensatory cortical strategies to accommodate for the inability to maintain the desired force levels due to peripheral fatigue. Supporting this, Liu et al. (2007) advocated that muscle fatigues well before the brain does; in essence that peripheral fatigue occurs before central fatigue. They demonstrated, by estimating the changes of source locations of high-density EEG signals using a single moving current dipole model, that handgrip muscle fatigue was associated with shifting of brain activation centers from one location to another when neurons in the previous location become fatigued. These studies collectively demonstrate the application of EEG in examining the neural correlates of localized fatigue development of smaller muscles during relatively static, or immobile, tasks.

Of the various neuroimaging techniques, EEG offers the greatest flexibility and mobility features that make it an attractive candidate in assessing whole body fatigue. By simultaneously obtaining information on eye movements and spontaneous EEG signals, Kubitz and Mott (1996) demonstrated increased brain activation (i.e., decreased alpha activity and increased beta activity) during a fatiguing cycling task. While technical advances have been made in minimizing mechanical artifacts from high-density EEG signals during whole body movements (Gwin et al., 2010), fNIRS has gained rapid attention in evaluating whole body fatigue owing to its methodological advantages over EEG. First, fNIRS provides information on the location of the neural signal generated, whereas with EEG signals, source localization has to be computationally derived. Second, there are no time-sensitive requirements in examining whole body fatigue when compared to fast reaction time tasks; slower hemodynamic responses of fNIRS are thus appropriate when compared to fast EEG responses. As such, fNIRS responses have shown to be less affected by movement artifacts than EEG signals (Perrey, 2008). Several fNIRS studies have reported a significant decrease in relative levels of oxygenated hemoglobin in the prefrontal cortex, accompanied by muscular impairment, at exhaustion during submaximal and maximal fatiguing contractions (González-Alonso et al., 2004; Bhambhani et al., 2007; Nybo and Rasmussen, 2007). In particular, Thomas and Stephane (2008) demonstrated that oxygenated hemoglobin levels in the prefrontal cortex during incremental cycling exercise increased in the early stages, but decreased markedly in the last stage until exhaustion. These findings imply that prefrontal cortex activation is associated with reduction in motor output at the cessation of exercise. However, these fatiguing tasks are accompanied by cardiorespiratory changes in the autonomic system that can affect fNIRS responses (Obrig et al., 1996). Depending on the research questions asked, such systemic influences on cerebral hemodynamic responses may be desired or undesired. Obrig and Villringer (2003) emphasize the importance of analyzing deoxygenated hemoglobin levels as an indicator of “neuronal activation” over the more commonly used oxygenated hemoglobin values. They argue that oxygenated hemoglobin levels are acceptable neuronal activity indicators when cerebral autoregulation is intact, i.e., cerebral blood flow is in homeostasis. Increases in oxygenated hemoglobin during exercise can be attributed not only to neuronal activation but also to exercise-induced increased blood flow to the brain, and as such a decrease in deoxygenated hemoglobin is the most valid parameter. Thus, neuroergonomic investigations of fatigue need to consider these systemic influences, and perhaps collect peripheral measurements such as arterial blood pressure and heart rate to ensure that appropriate inferences are made from fNIRS signals.

## COGNITIVE WORK

The field of human factors and ergonomics had its origins in time-and-motion studies conducted in the early 1900s. With the advent of World War II, increasing attention was paid to evaluation of human psychological processes during work performance, but the dominant approach was behaviorism, or stimulus-response psychology. The advent of the cognitive revolution in the late 1950s lead to the introduction of the cognitive approach in human performance assessment from the 1960s to the present day, but there was still a relative neglect of brain mechanisms. Advances in neuroimaging and related methods that lead to the development of the field of cognitive neuroscience lead to the argument that neural measures should also be considered in human factors and ergonomics (Parasuraman, 2003). Since that time, the neuroergonomic approach has been applied to a number of different issues in cognitive ergonomics.

These historical trends in theoretical frameworks used in ergonomics can be seen clearly in the periodical reviews of the field of engineering psychology in the *Annual Reviews of Psychology*. Fitts (1958) reviewed work conducted mainly within time-and-motion and stimulus-response frameworks; Wickens and Kramer (1985) presented a cognitive or information-processing approach; and the most recent review, by Proctor and Vu (2010), describes the neuroergonomic approach. In this paper, we review a few key issues in cognitive neuroergonomics and on those areas where the most research and development work has been done. These include: (1) mental workload, (2) vigilance and mental fatigue, and (3) neuroadaptive systems.

### MENTAL WORKLOAD

The assessment of human mental workload is one of the most widely studied topics in ergonomics (Wickens and McCarley, 2008). If operator mental workload is either too high or too low human-system performance may suffer in work environments, thereby potentially compromising safety. Hence, workload must be assessed in the design of new systems or the evaluation of existing ones. Behavioral measures, such as accuracy and speed of response on secondary tasks, or subjective reports (such as the NASA-TLX) have been widely used to assess mental workload. However, measures of brain function offer some unique advantages that can be exploited in mental workload assessment (Kramer and Parasuraman, 2007). Among these is the ability to extract covert physiological measures continuously in complex system operations in which overt behavioral measures may be relatively sparse.

The dominant theory of human mental workload is *resource* theory (Wickens, 1984, 2002). This theory postulates that except for highly overlearned “automatic” tasks, task performance is directly proportional to the application of attentional resources. The theory also proposes that the degree of overlap of multiple pools of resources determines the pattern and amount of interference when two or more tasks are performed simultaneously (such as driving and talking on the cell phone). Dual-task studies have provided abundant support for resource theory (Wickens and McCarley, 2008), but one criticism is that the theory is circular (Navon, 1984), which can be linked to the lack of an independent measure of resources. This criticism can be countered if neural measures of mental resources can be identified.

Measures of cerebral hemodynamics, such as fNIRS and TCDS, have provided validation for the resource construct. In a recent study, Ayaz et al. (2012) tested experienced air traffic controllers (ATC) on a complex ATC task requiring them to keep aircraft in their sector free of conflicts. fNIRS was used to measure activation of the prefrontal cortex. Ayaz et al. (2012) found there was an increase in prefrontal cortex activation as the number of aircraft in their sector increased. These findings suggest that fNIRS can provide a sensitive index of cognitive workload in a skilled group performing a realistic task that was highly representative of their work environment. fNIRS has also been found to index changes in prefrontal cortex activation with skill acquisition in both basic working memory tasks (McKendrick et al., 2014) and more complex piloting tasks (Ayaz et al., 2012). Most recently, portable versions of fNIRS have been developed for use in mobile neuroimaging (Ayaz et al., 2013).

There are many factors, such as cost, ease of implementation, intrusiveness, etc., that must be taken into consideration when selecting neuroergonomic techniques for mental workload assessment. Some of these factors (e.g., cost) may rule out the use of neuroergonomic methods in favor of simpler indexes such as subjective measures. Some workers may also not wish to be “wired up” for physiological recording, so operator acceptance must also be carefully considered. However, with increasing miniaturization and development of dry electrode, wireless, wearable systems, some of these concerns are diminishing.

### VIGILANCE AND MENTAL FATIGUE

The evaluation of operator vigilance and mental fatigue in work environments is a topic closely related to workload assessment. The widespread implementation of automation in many work environments, including air and surface transportation and health care, while often leading to a reduction in operator workload, can also increase workload because of the resulting need for monitoring the automation (Parasuraman, 1987). The typical finding in vigilance studies is that the detection rate of critical targets declines with time on task (Davies and Parasuraman, 1982). Vigilance decrement was originally attributed to a reduction in physiological arousal (Frankmann and Adams, 1962) but more recent neuroergonomic research using TCDS and fNIRS have attributed it to resource depletion (Warm et al., 2008). Warm et al. (2008) reported a series of studies of TCDS and vigilance (for reviews, see Warm and Parasuraman, 2007; Warm et al., 2008). A consistent finding is that the vigilance decrement is paralleled by a decline in blood flow velocity over time, relative to a baseline of activity just prior to beginning the vigilance session. The parallel decline in vigilance performance and in blood flow velocity is found for both visual and auditory tasks (Shaw et al., 2009). These findings have been interpreted using resource theory. A critical control finding in support of resource theory – as opposed to a generalized arousal or fatigue model – is that the blood flow change occurs only when observers actively engage with the vigilance task. When observers are asked to simply watch a display passively without having to detect targets for the same amount of time as in an active vigilance condition – a case of maximal under-arousal – blood flow velocity does not decline but remains stable over time.

The deleterious effects of loss of operator vigilance can countered with reduced work hours and more frequent rest breaks, but this may not be practical in all work settings. Another mitigating strategy is to use cueing. Detection performance in vigilance tasks can be improved by providing observers with consistent and reliable cues to the imminent arrival of critical signals, with the extent of the decrement being reduced or eliminated (Wiener and Attwood, 1968). With cueing, observers need to monitor a display only after having been prompted about the arrival of a signal and therefore can husband their information processing resources over time. In contrast, when no cues are provided, observers are never certain of when a critical signal might appear and consequently have to process information on their displays continuously across the watch, thereby consuming more of their resources over time than cued observers. If the vigilance decrement stems from resource depletion due to need to attend continuously to a display, then pre-cues should reduce the decline in cerebral blood flow velocity as measured by TCDS. This was confirmed in a study by Hitchcock et al. (2003). They used no pre-cues or pre-cues that were 100, 80, or 40% reliable in pointing to an upcoming critical event in a simulated air traffic control task. Performance efficiency remained stable when perfectly reliable cues were provides but declined over time in the remaining conditions, so that by the end of the vigil, performance efficiency was clearly best in the 100% group, followed in order by the 80, 40%, and no-cue groups. Blood flow declined in the no cue control condition, but there was a progressive reduction in the extent of the decline with progressively more reliable cues. There was no decline when the cues were perfectly reliable. This pattern of change in blood flow exactly matched that of performance.

In addition to cueing, non-invasive brain stimulation could also be used to mitigate vigilance decrement and mental fatigue. Nelson et al. (2014) applied 1 mA anodal tDCS to either the left or right prefrontal cortex while participants performed the same vigilance task used by Hitchcock et al. (2003). tDCS was applied either early or late during the course of the vigilance task. Compared to a control group that showed the normal vigilance decrement, the early stimulation group had a higher detection rate of critical signals. The late stimulation group initially exhibited a vigilance decrement, but this was reversed following application of tDCS. These initial findings are highly encouraging, but need to be followed up with additional research to examine the long-term effectiveness of tDCS as a method to alleviate vigilance problems at work.

### TRAINING AND NEUROADAPTIVE SYSTEMS

While the goal of ergonomic design is to avoid having workers exposed to extremes of workload and to loss of vigilance, this may not always be possible in certain work settings where unexpected events, equipment failures, or other unanticipated factors lead to a transient increase in the task load imposed on the human operator, or long work hours impose demands on operator vigilance. Adaptive automation offers one approach to deal with these issues (Parasuraman, 1987, 2000). In this approach, the allocation of functions to human and machine agents is flexible during system operations, with greater use of automation during high task load conditions or emergencies and less during normal operations, consistent with the approach of dynamic function allocation (Lintern, 2012). The adaptive automation concept has a long history (Parasuraman et al., 1992), but neuroergonomic methods for its implementation have been considered relatively recently (Inagaki, 2003; Parasuraman, 2003; Scerbo, 2007).

Several methods to implement adaptive systems have been examined, including neuroergonomic measures to assess the operator’s functional state (Byrne and Parasuraman, 1996; Kramer and Parasuraman, 2007; Wilson and Russell, 2007; Parasuraman and Wilson, 2008; Ting et al., 2010). Many studies have used EEG because of its ease of recording and (relative) unobtrusiveness (compared, say, to secondary tasks or subjective questionnaires). EEG also has the property of being a very high bandwidth measure, offering the possibility of sampling the human operator at up to about 30 Hz (Wilson and Russell, 2003). Workload adaptive systems need to assess operator state in real time, or near real time, so that task allocation or restructuring can be implemented in cases of overload or underload. A number of different statistical and machine learning techniques have been used for this purpose. These include discriminant analysis (Berka et al., 2004), artificial neural networks (Wilson and Russell, 2007; Baldwin and Penaranda, 2012), Bayesian networks (Wang et al., 2011), and fuzzy logic (Ting et al., 2010). These have been implemented in real time and typically provide accuracies of 70–85%.

Implementing neuroergonomic adaptive systems in real settings poses significant challenges. A major issue concerns the detection and removal of artifacts in real time. Furthermore, while initial success has been achieved in using computational techniques to classify workload on the basis of EEG and other neuroergonomic measures, the reliability and stability of these methods within and across individuals needs to be more rigorously tested (Wang et al., 2011; Christensen et al., 2012). Finally, the operational community must be involved in the design of adaptive systems to ensure user acceptance and compliance.

## NEUROERGONOMIC ASSESSMENT OF CONCURRENT PHYSICAL AND COGNITIVE WORK

Both physical and cognitive neuroergonomics have helped advance our understanding on the role of the human brain during physical and cognitive work, respectively. Only a small number of studies have investigated the interaction between physical and cognitive work, which is a big concern since “work” places combined physical and cognitive demands on operators, never either one in isolation. High cognitive demands can influence physical work; and physical activity can in turn influence cognitive processing. In comparison to traditional evaluation techniques in either physical or cognitive ergonomics domain, neuroergonomic methods offer a great advantage in assessing these combined demands. For example, using EEG signals Kamijo et al. (2004) investigated the influence of exercise intensity on cognitive function using the P300 ERP component. They suggested that exercise influenced the amount of attentional resources devoted to a given task and that the changes in P300 amplitude followed an inverted *U*-shaped behavior of differences in exercise intensity. When examining the impact of cognitive demand on physical capacity, a few studies have attributed decreased muscle endurance in presence of a cognitively stressful situation to lower motivation (Marcora et al., 2009), increased neuromotor noise impairing joint steadiness (van Loon et al., 2001; Mehta and Agnew, 2011, 2013; Mehta et al., 2012), or neuronal interference at the prefrontal cortex that is involved in cognitive processing and isometric motor contractions (Dettmers et al., 1996; Rowe et al., 2000; Mehta and Parasuraman, 2013). In particular, using fNIRS to monitor cerebral oxygenation during handgrip exercises, Mehta and Parasuraman (2013) demonstrated that concurrent handgrip exercises in cognitive stressful conditions were associated with lower oxygenated hemoglobin levels in the bilateral prefrontal cortex at exhaustion when compared to the handgrip exercises at the same intensity levels (i.e., no changes observed in peripheral muscular responses of EMG and force exerted) under no stress. Quite similarly, using EEG- and EMG-derived corticomuscular coupling measure, Kristeva-Feige et al. (2002) reported that corticomuscular coupling decreased significantly during a cognitively stressful condition despite no changes observed in traditional measures such as EMG and force production. These studies collectively emphasize the importance of obtaining brain (or central) responses along with the more conventional ergonomic measurements to accurately understand the “total” demands placed on humans during work that requires both physical and cognitive processing. Future investigations on comparing these brain-body responses with the more traditional performance or subjective measures are also needed to understand the underlying neural “cost” of operator functional state. Such studies are also needed so as to develop evaluation tools (surveys, heuristic checklists) that are predictive of the neural and physiological cost associated with optimizing work tasks, which can be used by designers or supervisors to quantify operator workload and fatigue.

## MOBILE BRAIN IMAGING CONSIDERATIONS FOR WORKLOAD/FATIGUE ASSESSMENTS

One of the key distinctions between neuroergonomics and neuroscience is that neuroergonomics is the study of brain and behavior “at work.” Thus, it is extremely important that neuroergonomic methods are capable of examining human operators at their naturalistic work settings. In this paper, we discussed the merits and disadvantages of the available neuroimaging techniques applicable to neuroergonomics and a key theme identified was the lack of studies evaluating neural bases of mobile work, particularly in the physical neuroergonomics domain. Recent efforts in developing mobile brain imaging (MoBI) techniques, which consider the physical and environmental impact on human cognitive processing, show great promise. For example, Gramann et al. (2011) reviewed the implications and feasibility of a newly developed MoBI system that was previously employed in examining cognitive processing during human stance and locomotion. In particular, their MoBI investigation included simultaneous brain-body measurements from a 256-channel EEG system and kinematic and kinetic outcomes that are otherwise employed during conventional gait biomechanics using motion capture systems and force plates (Gwin et al., 2011). In their review, Gramann and colleagues identify key requirements for MoBI methods that include: (1) robust mobile sensor technology to measure brain activity, (2) comprehensive “wireless” body measurement system, and (3) powerful computational software to collectively processing and analyze both brain-body responses. While developing an ideal MoBI system may be a challenging goal, understanding current limitations in mobile brain-body imaging and addressing them, albeit painstakingly, is a critical step toward achieving this goal. Future investigations can also include developing similar mobile brain-body imaging systems for hemodynamic neuroimaging techniques, utilizing either fNIRS or TCDS to provide brain imaging measures, and using peripheral measurements such as heart rate and blood pressure to document physiological whole-body responses.

## CONCLUSION

Ergonomics has long since moved from being a science of improving work efficiency to now being focused on enhancing well-being while improving systems performance. To effectively understand how humans interact with work systems, it is not only important to ask how well they perform, but also *why* they perform a certain way. Neuroergonomics have helped fill in the gaps on the neural bases of both physical and cognitive performance that were left unanswered with traditional ergonomic assessments. In this review we discussed the recent developments and adoption of neuroergonomic methods and applications in investigating physical, cognitive, and combined physical and cognitive work. We also reviewed the applicability and feasibility of neuroimaging techniques in evaluating mobile work environments. While some neuroimaging methods are expensive and are immobile, such as the MRI, fMRI, PET, and DTI, portable methods such as EEG, fNIRS, and TCDS, are more likely to be adopted in applied ergonomics research. With the advent of, and recent developments in, MoBI technology, we can be assured that neuroergonomics can continue providing critical information on how/why human interact in ambulatory and naturalistic work settings.

## AUTHOR CONTRIBUTIONS

Both authors contributed equally to this work. Ranjana K. Mehta performed the literature review on neuroergonomics applications to physical work and Raja Parasuraman performed the literature review on cognitive neuroergonomics. Both authors discussed the reviewed implications and commented on the manuscript at all stages.

## Conflict of Interest Statement

The authors declare that the research was conducted in the absence of any commercial or financial relationships that could be construed as a potential conflict of interest.
